# Application of the new classification proposal for juvenile idiopathic arthritis of the pediatric rheumatology international trials organization in a group of Mexican patients

**DOI:** 10.3389/fped.2024.1476257

**Published:** 2024-11-07

**Authors:** Pamela Ramos-Tiñini, Héctor Menchaca-Aguayo, Deshire Alpizar-Rodriguez, Esther Mercedes-Pérez, Enrique Faugier-Fuentes

**Affiliations:** ^1^ Pediatric Rheumatology Department, Hospital Infantil de México Federico Gómez, Mexico City, Mexico; ^2^ Research Unit, Colegio Mexicano de Reumatología, Mexico City, Mexico

**Keywords:** Juvenile idiopathic arthritis, classification, rheumatoid factor, antinuclear antibodies, ILAR, PRINTO

## Abstract

**Objective:**

Apply the PRINTO classification proposal for diagnosing Juvenile Idiopathic Arthritis (JIA) to Mexican patients, analyzing demographic, clinical, and laboratory characteristics.

**Material and methods:**

Cross-sectional study analyzing patients diagnosed with JIA using International League of Associations for Rheumatology (ILAR 2001) criteria over two years at a national rheumatic disease center. Reclassification was done using the Pediatric Rheumatology International Trials Organization (PRINTO) proposal. Comparisons were made between antinuclear antibodies (ANAs) positive vs. negative and rheumatoid factor (RF) positive vs. negative patients.

**Results:**

Seventy-six patients were analyzed, mostly female. Median age was lower in systemic JIA (sJIA) and early onset JIA with positive ANAs (eoANA JIA). ANAs was present in 78.6% of patients. Reclassification according to PRINTO disorders showed RF positive polyarticular JIA, sJIA, and enthesitis-related JIA (ER JIA) reclassified to RF JIA, sJIA, and enthesitis/spondylitis-related JIA (ESR JIA) by 100%, 94.7%, and 80%, respectively. The ILAR category with the most variation was RF negative polyarticular JIA. Early disease onset was associated with a lower probability of positive RF after adjusting for sex, age, and ANAs. No association was found between ANAs positive vs. negative in adjusted multivariate analysis.

**Conclusions:**

We found compatibility of sJIA, RF positive polyarticular JIA, and RE JIA categories with sJIA, RF JIA, and ESR JIA disorders, respectively. Differences were noted in variables such as sex and the number of affected joints. There was high ANAs positivity; however, few patients were classified into eoANA JIA disorder, with only one presenting uveitis. Most patients were classified as other JIA.

## Introduction

Juvenile Idiopathic Arthritis (JIA) is a heterogeneous group of diseases, encompassing various types of arthritis classified according to the International League of Associations for Rheumatology (ILAR). JIA is the most common chronic autoimmune disease in pediatrics and can cause both short and long-term disabilities (1). In 1972 and 1977, the American College of Rheumatology (ACR) and the European Alliance of Associations for Rheumatology (EULAR) proposed classification criteria. However, due to differences between these classifications, ILAR proposed a unified classification in 1994 (2). This classification has been reviewed twice, most recently in 2001 (3, 4). The widely used ILAR classification considers children under 16 years old who have had arthritis for at least 6 weeks and evaluates their clinical behavior during the first 6 months. Nevertheless, there are controversies in applying the criteria to certain subtypes. For instance, the systemic JIA (sJIA) subtype could be reclassified as an autoinflammatory disease due to its physiopathogenic characteristics (5). Additionally, some studies have reported the onset of arthritis following fever (6), leading to the proposal of using the Yamaguchi criteria (7). The enthesitis related JIA (ER JIA) subtype is part of the spondyloarthritis group, which is heterogeneous in terms of presentation, clinical behavior, and its association with HLA-B27 (8–10). Identifying psoriatic JIA (PsJIA) can be challenging since the presentation of psoriasis can be atypical, delaying diagnosis. It also shares clinical characteristics with oligoarticular JIA and ER JIA (11–13). These challenges have spurred the development of new criteria to classify patients more homogeneously. The Pediatric Rheumatology International Trials Organization (PRINTO) proposed a classification that considers genetic background and disease expression as a group of disorders with arthritis as a common denominator, regardless of the number of joints affected and applying ANAs, RF and anti-cyclic citrullinated peptide antibody (anti-CCP Ab), which have been proven to constitute a homogenous disease entity (14), unlike the ILAR classification. These criteria are currently undergoing validation in different populations but have not been applied to the Mexican population. The objective of our study is to apply the new PRINTO provisional classification to a group of Mexican patients with JIA from the Children's Hospital of Mexico Federico Gómez, who were previously classified according to ILAR, and to analyze the demographic, clinical, and laboratory characteristics present in each disorder.

## Materials and methods

### Patient and study outcomes

Cross-sectional study was conducted at the Children's Hospital of Mexico Federico Gómez, a leading pediatric hospital in Mexico City. It included all patients aged 0–16 years with a confirmed diagnosis of JIA according to the ILAR 2001 classification criteria, consecutively attended Pediatric Rheumatology consultations between 2019 and 2020. Exclusion criteria were loss to follow-up during this period and patients with incomplete information on the clinical file. The protocol was approved by the local ethics committee number HIM/SR/2024/002. Since this was a study comprising a review of de-identified data, written informed consent was not required.

The ILAR criteria define JIA as arthritis of unknown etiology beginning before age 16 and persisting for at least 6 weeks (3). Exclusion criteria are used to differentiate the seven subtypes: systemic, oligoarticular, RF positive polyarticular, RF negative polyarticular, enthesitis related, psoriatic, and undifferentiated (Supplementary Table S1) (3). Patients were reclassified based on the new PRINTO classification proposal, which considers JIA as a group of inflammatory disorders that begin before age 18 and persist for at least 6 weeks, excluding other known conditions (Supplementary Table S2) (14).

Data were collected from clinical records, including clinical manifestations and laboratory studies. A single data collection form was used, recording the following information: sex, age, age at symptom onset and diagnosis, time of presentation, symptoms, laboratory tests including immunological studies (ANAs, anti-CCP Ab, RF, and HLA-B27). RF was measured by immunonephelometry and considered positive (≥15 IU/ml). ANAs were tested by immunofluorescence and considered positive with titers ≥1:160. Anti-CCP Ab was measured by immunoassay, and HLA-B27 by flow cytometry was considered positive based on the reference value.

### Statistical analysis

The information was recorded in a database, and a descriptive analysis of variables was performed. Measures of central tendency and dispersion were calculated using medians and interquartile ranges for quantitative variables, and frequencies for nominal and ordinal variables. Student's *t*-test or Mann-Whitney or and Chi-square or Fisher's exact test were used to compare groups as appropriate. Univariable and multivariable logistic regression analyses were performed to compare associations between variables and ANAs positive vs. ANAs negative status and RF positive vs. RF negative status. Statistical significance was considered less than 0.05. All analyses were performed using STATA 14.0 (Stata Corp LP, College Station, TX, USA).

## Results

**Figure 1:**
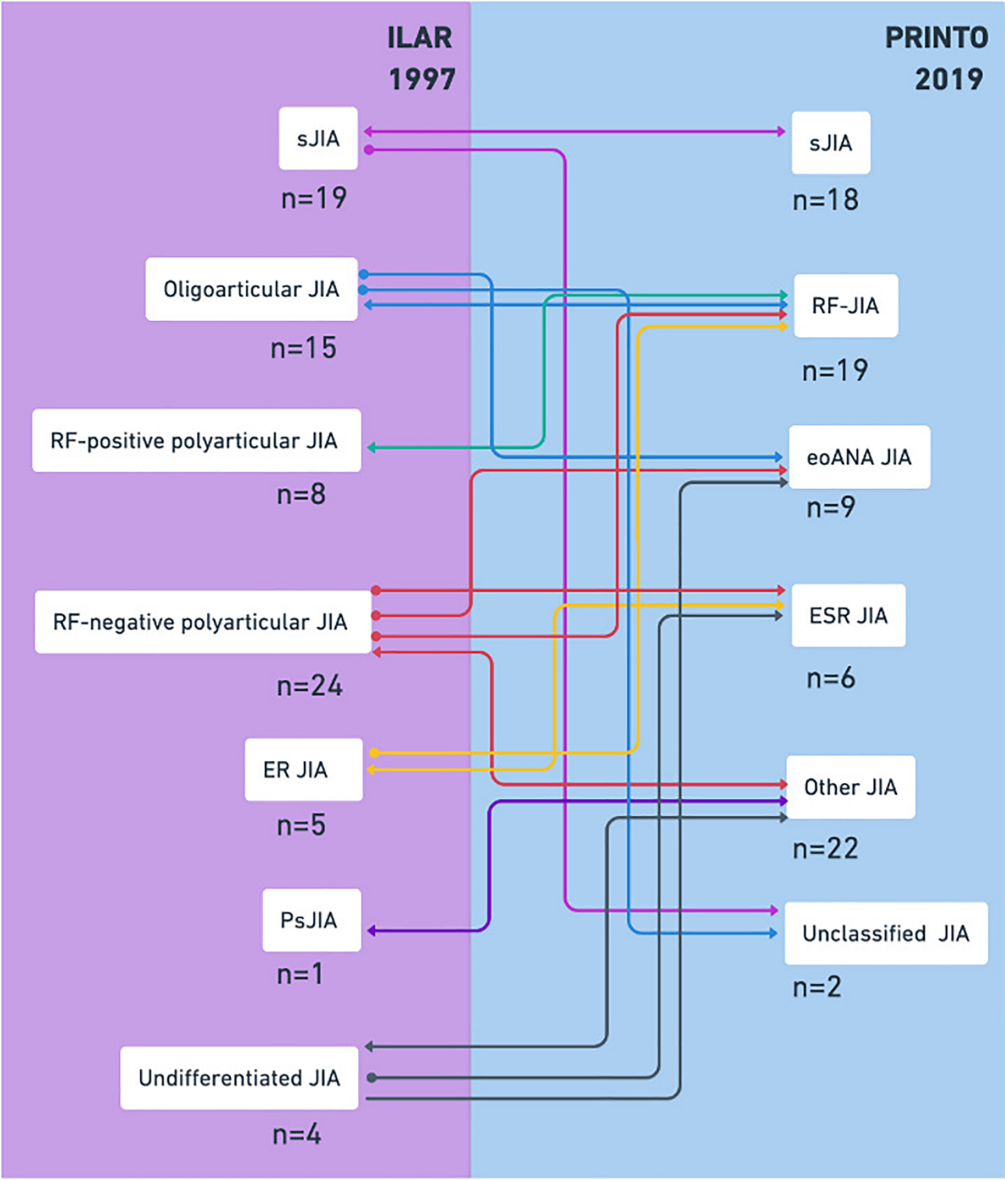
Application of classification criteria in JIA. The reclassification of ILAR categories to PRINTO disorders is shown schematically. JIA, Juvenile idiopathic arthritis; ANAs, antinuclear antibodies; RF, rheumatoid factor; sJIA, systemic JIA; ER JIA, enthesitis-related JIA; Ps JIA, psoriatic JIA; eoANA JIA, early onset JIA with positive ANAs; ESR JIA, enthesitis/spondylitis-related JIA.

A total of 79 patients diagnosed with JIA according to the ILAR criteria were included. Three patients were excluded: two due to loss to follow-up and one that did not meet the ILAR criteria. Thus, 76 patients were analyzed (Tables 1, 2), with a female predominance (60.5%). The median age was lower in patients with sJIA and eoANA JIA disorders, both at 4.7 years. Among patients, 98.7% had no family history of chronic arthritis in first-degree relatives. In addition to eoANA JIA disorder, which considers early onset for classification, sJIA disorder predominantly presented in children younger than 6 years old, accounting for 72.2%. All patients had arthritis at diagnosis, with polyarthritis being the most common presentation (78.9%). In the sJIA disorder, all patients had fever and arthritis at diagnosis; 83.3% had the characteristic rash included in the major criteria of the new classification, and hepatomegaly was the predominant minor criterion (66.7%). In ESR JIA disorder, all patients had peripheral arthritis, with sacroiliac tenderness in 83.3%, and both enthesitis and lumbosacral pain in 50%. Within the RF JIA disorder, 94.7% had positive RF, and one patient was anti-CCP Ab positive, included in the new classification. Over half (57.9%) had leukocyte levels between 5,000 and 10,000 mm³ at diagnosis. Reclassifying patients from ILAR to PRINTO disorders (Figure 1), ILAR categories RF positive polyarticular JIA, sJIA, and ER JIA were reclassified to RF JIA, sJIA, and ESR JIA disorders at rates of 100%, 94.7%, and 80%, respectively. The ILAR category with the most variation was RF negative polyarticular JIA, reclassified to Other JIA (62.5%) and eoANA JIA (29.2%) disorders. Among the total sample, 23.7% had positive RF (Table 3). Comparing patients with positive vs. negative RF, ANAs positivity was associated with positive RF, and disease onset before age 6 was linked to a lower likelihood of positive RF. In multivariable analysis adjusted for sex, age, and ANAs, the only persistent association was age under 6 years (OR 0.2, 95% CI 0.05–0.83). Of the patients who were tested for ANAs, 78.6% were positive, but only 9 (12.9%) were reclassified into eoANA JIA disorder. Univariate analysis showed female gender, RF, and enthesitis were associated with positive ANAs (Table 4). No associations persisted in multivariate analysis. In the adjusted sub-analysis of ANAs with sex and age, no associations were found in the 31 patients with eoANA JIA and Other JIA.

**Table 1:** PRINTO disorders.

	sJIA *N* = 18	RF JIA *N* = 19	ESR JIA *N* = 6	eoANA JIA *N* = 9	Other JIA *N* = 22	Unclassified JIA *N* = 2
Gender (female), *n* (%)	10 (55.5)	14 (73.6)	5 (83.3)	2 (22.2)	14 (63.7)	1 (50)
Age, median (IQR)	4.7 (4.1–7.6)	8.2 (6.4–10.8)	6.0 (4.5–9.6)	4.7 (3.1–5.0)	8.7 (6.8–10.0)	7.3 (4.7 −9.8)
Time to diagnosis (<6 months), *n* (%)	8 (44.4)	9 (47.4)	1 (16.7)	5 (55.6)	10 (45.4)	2 (100)
Family history, *n* (%)	1 (5.6)	0	0	0	0	0
Early onset, *n* (%)	13 (72.2)	2 (10.5)	2 (33.3)	9 (100)	5 (22.7)	1 (50)
Symptoms						
Arthritis, *n* (%)	18 (100)	19 (100)	6 (100)	9 (100)	22 (100)	2 (100)
Fever, *n* (%)	17 (94.4)	0	0	0	0	1 (50)
Evanescent exanthema, *n* (%)	15 (83.3)	0	0	0	0	0
Lymphadenopathy, *n* (%)	9 (50.0)	1 (5.3)	0	0	0	1 (50)
Hepatomegaly, *n* (%)	12 (66.7)	0	0	0	0	1 (50)
Splenomegaly, *n* (%)	5 (27.8)	0	0	0	0	1 (50)
Serositis, *n* (%)	1 (5.6)	0	0	0	0	0
Polyarthritis, *n* (%)	17 (94.4)	14 (73.7)	3 (50.0)	7 (77.8)	17 (77.3)	2 (100)
Oligoarthritis, *n* (%)	1 (5.6)	5 (26.3)	1 (16.7)	1 (11.1)	5 (22.7)	0
Enthesitis, *n* (%)	0	0	3 (50)	0	0	1 (50)
Dactilitis, *n* (%)	0	1 (5.3)	0	0	0	0
Sacroiliac tenderness, *n* (%)	0	2 (10.5)	5 (83.3)	1 (11.1)	1 (4.5)	1 (50)
Lumbosacral pain, *n* (%)	0	2 (10.2)	3 (50.0)	0	0	0
Acute uveitis, *n* (%)	0	1 (5.3)	1 (16.7)	0	0	0
Psoriasis, *n* (%)	0	0	0	0	1 (4.5)	0
Nail involvement, *n* (%)	0	0	0	0	0	0
Arthralgia, *n* (%)	18 (100)	19 (100)	6 (100)	9 (100)	22 (100)	2 (100)
Laboratory tests						
RF, *n* (%)^a^	0	18 (94.7)	0	0	0	1 (50)
HLA_B27, *n* (%)^b^	0	0	1 (16.7)	1 (11.1)	0	0
Leukocytes <5,000, *n* (%)	0	0	0	1 (11.1)	1 (4.5)	0
Leukocytes 5–10 mil, *n* (%)	7 (38.9)	16 (84.2)	3 (50)	6 (66.7)	10 (45.5)	2 (100)
Leukocytes 10–15 mil, *n* (%)	2 (11.1)	5 (26.3)	2 (33.3)	0	7 (31.8)	0
Leukocytes >15 mil, *n* (%)	9 (50)	1 (5.3)	1 (16.7)	1 (11.1)	2 (9.1)	0
Ant-CCP, *n* (%)^c^	1 (5.6)	4 (21.1)	0	0	1 (4.5)	0
ANA, *n* (%)^d^	11 (61.1)	18 (94.7)	1 (16.7)	9 (100)	14 (63.6)	2 (100)

^a^ 74 patients.

^b^ 16 patients.

^c^ 17 patients.

^d^ 70 patients.

**Table 2:** ILAR categories and reclassification in PRINTO.

ILAR Categories							
PRINTO disorders	sJIA *n* = 19	RF negative polyarticular JIA *n* = 24	RF positive polyarticular JIA *n* = 8	Oligoarticular JIA *n* = 15	ER JIA *n* = 5	PsJIA *n* = 1	Undifferentiated JIA *n* = 4
sJIA, 18 (%)	18 (94.7)	0	0	0	0	0	0
RF JIA, 19 (%)	0	1 (4.2)	8 (100)	9 (60)	1 (20)	0	0
ESR JIA, 6 (%)	0	1 (4.2)	0	0	4 (80)	0	1 (25)
eoANA JIA, 9 (%)	0	7 (29.1)	0	1 (6.7)	0	0	1 (25)
Other JIA, 22 (%)	0	15 (62.5)	0	4 (26.7)	0	1 (100)	2 (50)
Unclassified JIA, 2 (%)	1 (5.3)	0	0	1 (6.7)	0	0	0

It numerically shows the patients classified into ILAR categories and their reclassification in PRINTO.

**Table 3:** Univariable and multivariable logistic regression analyses on RF.

	RF positive 18 (24.3%)	RF negative 56 (75.7%)	*p*
Female, *n* (%)	14 (77.8)	31 (55.4)	0.43
Age ≤6 years, *n* (%)	2 (11.1)	26 (46.4)	**0.004**
Uveitis, *n* (%)	0	2 (3.6)	0.39
Enthesitis, *n* (%)	1 (5.6)	3 (5.4)	0.97
ANA, *n* (%) *n* = 70	18 (100)	37 (66.1)	**0.01**
HLA-B27 *n* = 16	0	2 (3.6)	0.69
Oligoarthritis *n* (%)	4 (22.2)	9 (16.1)	0.64
Polyarthritis *n* (%)	14 (77.8)	45 (80.4)	0.44

Bold values indicate statistically significant results.

**Table 4:** Univariable and multivariable logistic regression analyses on ANAs.

	ANAs positive 55 (78.6)	ANAs negative 15 (21.4)	*p*
Female, *n* (%)	30 (54.6)	13 (86.7)	**0.02**
Age at disease onset, median (IQR)	7.1 (4.8–10)	6.5 (4.8–9.9)	0.79
Age ≤6 years, *n* (%)	19 (34.5)	7 (46.7)	0.36
Uveitis, *n* (%)	1 (1.8)	1 (6.7)	0.35
Enthesitis, *n* (%)	1 (1.8)	3 (20)	**0.0007**
HLA-B27, *n* (%) *n* = 16	1 (1.8)	1 (6.7)	0.54
Oligoarthritis *n* (%)	10 (18.2)	3 (20)	0.87
Polyarthritis *n* (%)	43 (78.2)	12 (80)	0.87
RF *n* (%)	18 (32.7)	1 (6.7)	**0.03**

Bold values indicate statistically significant results.

## Discussion

In this study, we applied the new PRINTO classification proposal to patients previously diagnosed with JIA according to the ILAR criteria. We analyzed the demographic, clinical, and laboratory characteristics. In this study, 18 out of 19 patients previously classified under the sJIA category were reclassified into the sJIA disorder, representing 23.7% of the total. This contrasts with the results of similar studies conducted in Canada and Portugal, where the sJIA disorder represented a smaller percentage, specifically 3.8% and 3%, respectively (15, 16). It is important to mention that, when applying the ILAR criteria, patients who did not present arthritis at the onset of the disease were excluded. This is one of the significant changes in the new proposal, as some patients may exhibit systemic symptoms prior to the presence of arthritis (6).

In the FR JIA disorder, all the patients corresponding to the polyarticular RF positive JIA category were reclassified, and 9 patients (60%) from the JIA oligoarticular category were added. Therefore, the new classification considers that the number of affected joints does not define a homogeneous subgroup of JIA. The increase in joint involvement at the time of diagnosis and over time reflects a faster evolution of arthritis in this disease. This highlights the importance of RF and anti-CCP Ab as immunological markers that determine the phenotype of this disorder (17, 18). Under this consideration, the number of affected joints may cause a single patient being classified into different ILAR categories throughout their life. Additionally, the physical examination may underestimate the presence of subclinical arthritis, which can be observed in imaging studies (19). The literature describes a female predominance and a later age of onset of the disease in this disorder (14). In line with these findings, where 73.7% of the patients were female, and in 89.5% of the cases, the disease began after the age of 6. Anti-CCP Ab was not measured in all patients due to limited availability and economic constraints that restricted implementation. The ESR JIA accounted for 80% of the patients in the RE JIA category, which is characterized by arthritis and enthesitis, with the latter in 50% of the total patients. Additionally, sacroiliac tenderness was observed in 83.3% of the cases. Imaging studies were a criterion in the new proposal; however, they were not performed on all patients because they are not required by the ILAR criteria, as the involvement is mainly peripheral in pediatric age (14). Acute uveitis, a common finding in this disorder, was present in 16.7% of the total, aligning with the expected one-fifth prevalence (20), Unlike typical cases with a male predominance and a family history of spondyloarthritis (21), our study found a predominance of females (83.3%) and no family history. HLA-B27 was not measured in all patients due to economic constraints. Regarding eoANA-JIA disorder, several studies have reported homogeneous characteristics such as early onset, female predominance, asymmetric arthritis, development of iridocyclitis, fewer affected joints over time, and absence of hip involvement (22). However, this study revealed a male predominance, polyarthritis, and no cases of uveitis were diagnosed. The Other JIA disorder includes those cases of arthritis that do not fit into the four main disorders, being the group in which most patients were classified (29%). These results are consistent with other similar studies, where the proportions of patients classified in this disorder were 39.5%, 63%, and 69.5%, respectively (15, 16, 23). Although other JIA does not represent a homogenous disease group by definition (14), of the 22 patients classified in this disorder, the majority belonged to the RF negative polyarticular JIA category (68.2%), this is in accordance with other studies where 66% and 78% of this category were reclassified in this disorder (15, 16). The unclassified JIA disorder includes patients who may be classified into more than one of the first four disorders; only two patients were classified in this disorder. As we can observe, we found high compatibility between the categories sJIA, polyarticular RF positive JIA and RE JIA with the sJIA, RF JIA, and ESR JIA disorders, similar to findings from other studies comparing ILAR and PRINTO classifications in different populations (15, 16, 23, 24). Different cohorts have reported varying rates of ANAs positivity in JIA. In two studies of Korean patients with JIA, ANAs positivity were found in 18% and 33% of the cases (25, 26). Conversely, studies conducted in India and Costa Rica reported significantly lower rates, with 1.1% and 6.3%, respectively (27, 28). This contrasts with our results, where 78.6% of the patients showed ANAs positivity, and only one patient developed uveitis. However, there are no comparative reports from Mexico. Additionally, we observed a higher RF positivity rate (24.3%) compared to other studies, which report a lower percentage ranging from 2% to 12% (29). This could imply a worse prognosis in our population. Univariable analysis of ANAs and RF positivity revealed a significant association. Although both antibodies distinguish between two different groups of disorders, there is evidence of peripheral helper T cells in the synovial tissue of patients with eoANA JIA, similar to those found in seropositive rheumatoid arthritis. This finding could imply the presence of pathogenic autoantibodies (30). Finally, a study comparing ANAs positivity in eoANA JIA and other JIA disorders, excluding sJIA, RF JIA, and ESR JIA due to their characteristics independent of ANAs positivity, found an association with female sex and the risk of uveitis (24). However, when performing this sub-analysis in our study, no significant association was found.

The limitations of this study include a small number of patients, the cross-sectional design, being conducted at a single center, and during the COVID-19 pandemic. We did not include patients aged 17 and 18 as per the new PRINTO proposal, as our center only attends to patients under 16, which reduced the number of participants. Additionally, not all patients had anti-CCP Ab and HLA-B27 tests, which are essential for the new classification. Further large-scale, prospective, and multi-ethnic studies are needed.

## Conclusion

This is one of the largest studies conducted in Latin American center and the first in an exclusively Mexican population, providing valuable information on this new classification proposal. We found a compatibility of the categories sJIA, RF positive polyarticular JIA and RE JIA with sJIA, RF JIA, and ESR JIA disorders, respectively, and differences were observed in variables such as sex and the number of affected joints. sJIA disorder included a larger number of patients. We found higher RF positivity, which could imply a worse prognosis in our population. We emphasize the importance of ANAs, RF, anti-CCP Ab as immunologic markers that constitute a homogeneous group. Although the study was conducted in patients with high ANAs positivity, a small number of patients were classified into eoANA-JIA disorder, only one patient presented uveitis. Finally, the majority of patients were classified as other JIA, highlighting the need for studies with a larger number of patients and in different ethnic groups to establish a more accurate classification system.

## Data Availability

The original contributions presented in the study are included in the article/Supplementary Material, further inquiries can be directed to the corresponding author.

## References

[1] Ravelli A Martini A. Juvenile idiopathic arthritis. Lancet. (2007) 369(9563):767–78. 10.1016/S0140-6736(07)60363-8 17336654

[2] Fink CW. Proposal for the development of classification criteria for idiopathic arthritides of childhood. J Rheumatol. (1995) 22(8):1566–9. 7473484

[3] Petty RE Southwood TR Manners P Baum J Glass DN Goldenberg J International league of associations for rheumatology classification of juvenile idiopathic arthritis: second revision, Edmonton (2001). J Rheumatol. (2004) 31(2):390–2. 14760812

[4] Petty RE Southwood TR Baum J Bhettay E Glass DN Manners P Revision of the proposed classification criteria for juvenile idiopathic arthritis: Durban, 1997. J Rheumatol. (1998) 25(10):1991–4. 9779856

[5] Nigrovic PA Raychaudhuri S Thompson SD. Review: genetics and the classification of arthritis in adults and children. Arthritis Rheumatol. (2018) 70(1):7–17. 10.1002/art.40350 29024575 PMC5805142

[6] Application of the yamaguchi criteria for classification of “suspected” systemic juvenile idiopathic arthritis (sJIA). *Pediatr Rheumatol*. Disponible en: https://ped-rheum.biomedcentral.com/articles/10.1186/1546-0096-10-40 (citado el 5 de Marzo de 2024). 10.1186/1546-0096-10-40 PMC3551717 23176399

[7] Yamaguchi M Ohta A Tsunematsu T Kasukawa R Mizushima Y Kashiwagi H Preliminary criteria for classification of adult still's disease. J Rheumatol. (1992) 19(3):424–30. 1578458

[8] Tse SML Laxer RM. New advances in juvenile spondyloarthritis. Nat Rev Rheumatol. (2012) 8(5):269–79. 10.1038/nrrheum.2012.37 22487801

[9] Adrovic A Sezen M Barut K Sahin S Acikel C Demirkaya E The performance of classification criteria for juvenile spondyloarthropathies. Rheumatol Int. (2017) 37(12):2013–8. 10.1007/s00296-017-3837-8 29018906

[10] Adrovic A Barut K Sahin S Kasapcopur O. Juvenile spondyloarthropathies. Curr Rheumatol Rep. (2016) 18(8):55. 10.1007/s11926-016-0603-y 27402112

[11] Egeberg A Skov L Zachariae C Gislason GH Thyssen JP Mallbris L. Duration of psoriatic skin disease as risk factor for subsequent onset of psoriatic arthritis. Acta Derm Venereol. (2018) 98(6):546–50. 10.2340/00015555-2912 29487945

[12] Naddei R Rebollo-Giménez A Burrone M Natoli V Rosina S Consolaro A Juvenile psoriatic arthritis: myth or reality? An unending debate. J Clin Med. (2023) 12(1):367. 10.3390/jcm12010367 36615167 PMC9821505

[13] Morris A Rogers M Fischer G Williams K. Childhood psoriasis: a clinical review of 1262 cases. Pediatr Dermatol. (2001) 18(3):188–98. 10.1046/j.1525-1470.2001.018003188.x 11437997

[14] Martini A Ravelli A Avcin T Beresford MW Burgos-Vargas R Cuttica R Toward new classification criteria for juvenile idiopathic arthritis: first steps, pediatric rheumatology international trials organization international consensus. J Rheumatol. (2019) 46(2):190–7. 10.3899/jrheum.180168 30275259

[15] Lee JJY Eng SWM Guzman J Duffy CM Tucker LB Oen K A comparison of international league of associations for rheumatology and pediatric rheumatology international trials organization classification systems for juvenile idiopathic arthritis among children in a Canadian arthritis cohort. Arthritis Rheumatol. (2022) 74(8):1409–19. 10.1002/art.42113 35289119

[16] Catarino S Nunes J Ganhão S Aguiar F Rodrigues M Brito I. Application of the new PRINTO classification criteria for juvenile idiopathic arthritis in a sample of Portuguese patients. ARP Rheumatol. (2024) 3(1):11–7. 10.63032/JXND6393 38558063

[17] Martini A. Are the number of joints involved or the presence of psoriasis still useful tools to identify homogeneous disease entities in juvenile idiopathic arthritis? J Rheumatol. (2003) 30(9):1900–3. 12966587

[18] Martini A. It is time to rethink juvenile idiopathic arthritis classification and nomenclature. Ann Rheum Dis. (2012) 71(9):1437–9. 10.1136/annrheumdis-2012-201388 22679300

[19] Magni-Manzoni S Epis O Ravelli A Klersy C Veisconti C Lanni S Comparison of clinical versus ultrasound-determined synovitis in juvenile idiopathic arthritis. Arthritis Rheum. (2009) 61(11):1497–504. 10.1002/art.24823 19877100

[20] Mistry RR Patro P Agarwal V Misra DP. Enthesitis-related arthritis: current perspectives. Open Access Rheumatol. (2019) 11:19–31. 10.2147/OARRR.S163677 30774484 PMC6354696

[21] Martini A Lovell DJ Albani S Brunner HI Hyrich KL Thompson SD Juvenile idiopathic arthritis. Nat Rev Dis Primers. (2022) 8(1):5. 10.1038/s41572-021-00332-8 35087087

[22] Ravelli A Varnier GC Oliveira S Castell E Arguedas O Magnani A Antinuclear antibody-positive patients should be grouped as a separate category in the classification of juvenile idiopathic arthritis. Arthritis Rheum. (2011) 63(1):267–75. 10.1002/art.30076 20936630

[23] Shoop-Worrall SJW Macintyre VG Ciurtin C Cleary G McErlane F Wedderburn LR Overlap of international league of associations for rheumatology and preliminary pediatric rheumatology international trials organization classification criteria for nonsystemic juvenile idiopathic arthritis in an established UK multicentre inception cohort. Arthritis Care Res (Hoboken). (2024) 76(6):831–40. 10.1002/acr.25296 38212149

[24] Kwon HJ Bang MH Kim KN. New provisional classification of juvenile idiopathic arthritis applying rheumatoid factor and antinuclear antibody. J Rheumatic Dis. (2018) 25(1):34–46. 10.4078/jrd.2018.25.1.34

[25] Shin JI Kim KH Chun JK Lee TJ Kim KJ Kim HS Prevalence and patterns of anti-nuclear antibodies in Korean children with juvenile idiopathic arthritis according to ILAR criteria. Scand J Rheumatol. (2008) 37(5):348–51. 10.1080/03009740801998762 18666025

[26] Lee JH Ryu JM Park YS. Clinical observations of juvenile rheumatoid arthritis. Clin Exp Pediatr. (2006) 49(4):424–30. 10.3345/kjp.2006.49.4.424

[27] Aggarwal A Misra R. Juvenile chronic arthritis in India: is it different from that seen in western countries? Rheumatol Int. (1994) 14(2):53–6. 10.1007/BF00300247 7529938

[28] Arguedas O Fasth A Andersson-Gäre B Porras O. Juvenile chronic arthritis in urban san José, Costa Rica: a 2 year prospective study. J Rheumatol. (1998) 25(9):1844–50. 9733470

[29] Borchers AT Selmi C Cheema G Keen CL Shoenfeld Y Gershwin ME. Juvenile idiopathic arthritis. Autoimmun Rev. (2006) 5(4):279–98. 10.1016/j.autrev.2005.09.011 16697970

[30] Nigrovic PA Colbert RA Holers VM Ozen S Ruperto N Thompson SD Biological classification of childhood arthritis: roadmap to a molecular nomenclature. Nat Rev Rheumatol. (2021) 17(5):257–69. 10.1038/s41584-021-00590-6 33731872 PMC10355214

