# Supplementary material for: Application of the new classification proposal for juvenile idiopathic arthritis of the pediatric rheumatology international trials organization in a group of Mexican patients
Source: Front Pediatr. 2024 Nov 7;12:1476257. doi: 10.3389/fped.2024.1476257 (PMC11578732; doi:10.3389/fped.2024.1476257)
Supplement: Supplementary file 1 [file Table1.docx]

**Supplementary Table 1. International League of Associations for Rheumatology Classification of Juvenile Idiopathic Arthritis: Second Revision, Edmonton, 2001(3).**

| **General Definition of JIA**  Juvenile idiopathic arthritis is arthritis of unknown etiology that begins before the 16th birthday and persists for at least 6 weeks; other known conditions are excluded. |
| --- |
| **Exclusions**  The principle of this classification is that all categories of JIA are mutually exclusive. This principle is reflected in the list of possible exclusions for each category:  a. Psoriasis or a history of psoriasis in the patient or first- degree relative.  b. Arthritis in an HLA-B27 positive male beginning after the 6th birthday.  c. Ankylosing spondylitis, enthesitis related arthritis, sacroiliitis with inflammatory bowel disease, Reiter’s syndrome, or acute anterior uveitis, or a history of one of these disorders in a first-degree relative.  d. The presence of IgM rheumatoid factor on at least 2 occasions at least 3 months apart.  e. The presence of systemic JIA in the patient.  The application of exclusions is indicated under each category, and may change as new data become available. |
| **Categories** |
| **Systemic Arthritis** *Definition:* Arthritis in one or more joints with or preceded by fever of at least 2 weeks’ duration that is documented to be daily (“quotidian”) for at least 3 days, and accompanied by one or more of the following:  1. Evanescent (nonfixed) erythematous rash 2. Generalized lymph node enlargement 3. Hepatomegaly and/or splenomegaly 4. Serositis  *Exclusions:* a, b, c, d. |
| **Oligoarthritis**  *Definition:* Arthritis affecting one to 4 joints during the first 6 months of disease. Two subcategories are recognized:  1. Persistent oligoarthritis: Affecting not more than 4 joints throughout the disease course  2. Extended oligoarthritis: Affecting a total of more than 4 joints after the first 6 months of disease *Exclusions:* a, b, c, d, e. |
| **Polyarthritis (Rheumatoid Factor Negative)**  *Definition:* Arthritis affecting 5 or more joints during the first 6 months of disease; a test for RF is negative. *Exclusions:* a, b, c, d, e. |
| **Polyarthritis (Rheumatoid Factor Positive)**  *Definition:* Arthritis affecting 5 or more joints during the first 6 months of disease; 2 or more tests for RF at least 3 months apart during the first 6 months of disease are posi- tive.  *Exclusions:* a, b, c, e. |
| **Psoriatic Arthritis**  *Definition:* Arthritis and psoriasis, or arthritis and at least 2 of the following:  1. Dactylitis 2. Nail pitting or onycholysis 3. Psoriasis in a first-degree relative  *Exclusions:* b, c, d, e. |
| **Enthesitis Related Arthritis**  *Definition:* Arthritis and enthesitis, or arthritis or enthesitis with at least 2 of the following:  1. The presence of or a history of sacroiliac joint tender- ness and/or inflammatory lumbosacral pain  2. The presence of HLA-B27 antigen 3. Onset of arthritis in a male over 6 years of age 4. Acute (symptomatic) anterior uveitis 5. History of ankylosing spondylitis, enthesitis related  arthritis, sacroiliitis with inflammatory bowel disease, Reiter’s syndrome, or acute anterior uveitis in a first-degree relative *Exclusions:* a, d, e. |
| **Undifferentiated Arthritis**  *Definition:* Arthritis that fulfills criteria in no category or in 2 or more of the above categories. |
