# Supplementary material for: Application of the new classification proposal for juvenile idiopathic arthritis of the pediatric rheumatology international trials organization in a group of Mexican patients
Source: Front Pediatr. 2024 Nov 7;12:1476257. doi: 10.3389/fped.2024.1476257 (PMC11578732; doi:10.3389/fped.2024.1476257)
Supplement: Supplementary file 2 [file Table2.docx]

**Supplementary table 2. Toward New Classification Criteria for Juvenile Idiopathic Arthritis: First Steps, Pediatric Rheumatology International Trials Organization International Consensus (14).**

| **General definition of JIA**. JIA comprises a group of inflammatory disorders that begins before the 18th birthday and persists for at least 6 weeks (must fulfill 1 of the A–E criteria below); other known conditions are excluded. |
| --- |
| **A. Systemic JIA: definition**. Fever of unknown origin (excluding infectious, neoplastic, autoimmune, or monogenic autoinflammatory diseases) that is documented to be daily (quotidian; fever that rises to ≥ 39°C once a day and returns to ≤ 37 °C between fever peaks) for at least 3 consecutive days and reoccurring over a duration of at least 2 weeks and accompanied by 2 major criteria OR 1 major criterion and 2 minor criteria. Major criteria are (1) evanescent (nonfixed) erythematous rash; and (2) arthritis. Minor criteria are (1) generalized lymph node enlargement and/or hepatomegaly and/or splenomegaly; (2) serositis; (3) arthralgia lasting 2 weeks or longer (in the absence of arthritis); and (4) leukocytosis (≥ 15,000/mm3) with neutrophilia. |
| **B. RF-positive JIA.**  - Arthritis for ≥ 6 weeks, and  - Association with 2 positive tests for RF at least 3 months apart or at least 1 positive test for antibodies to cyclic citrullinated peptide (CCP). |
| **C. Enthesitis/spondylitis-related JIA.**  - Peripheral arthritis and enthesitis, or  - Arthritis or enthesitis, plus ≥ 3 months of inflammatory back pain and sacroiliitis on imaging, or  - Arthritis or enthesitis plus 2 of the following: (1) sacroiliac joint tenderness; (2) inflammatory back pain; (3) presence of HLA-B27 antigen; (4) acute (symptomatic) anterior uveitis; and (5) history of a SpA in a first-degree relative.  If peripheral arthritis is present, it should persist for at least 6 weeks. |
| **D. Early-onset ANA-positive JIA.**  - Arthritis for ≥ 6 weeks, and  - Early-onset (≤ 6 yrs), and  - Presence of 2 positive ANA tests with a titer ≥ 1/160 (tested by immunofluorescence) at least 3 months apart.  Exclusions are systemic JIA, RF-positive arthritis, and enthesitis/spondylitis-related JIA. |
| **E. Other JIA.**  - Arthritis for ≥ 6 weeks  - Does not fit criteria for disorders A to D. |
| **F. Unclassified JIA.**  **-** Arthritis for ≥ 6 weeks  - Fits > 1 disorder A–D. |
